# Return to Sport After Acromioclavicular Injury: A Systematic Review of Modifiable Factors

**DOI:** 10.3390/jcm14217656

**Published:** 2025-10-28

**Authors:** William Chad Elliott, Benjamin Olivo, Alexander Abraham, Evan J. Hernandez, Tammam Hanna

**Affiliations:** 1 Texas Tech University Health Sciences Center School of Medicine, Lubbock, TX 79430, USA; chad.elliott@ttuhsc.edu (W.C.E.); benjamin.olivo@ttuhsc.edu (B.O.); 2 Department of Orthopaedic Surgery and Rehabilitation, Texas Tech University Health Sciences Center, Lubbock, TX 79430, USA; alexander.abraham@ttuhsc.edu (A.A.); evan.j.hernandez@ttuhsc.edu (E.J.H.); 3 Department of Health Sciences, College of Health Sciences, Rush University, Chicago, IL 60612, USA

**Keywords:** acromioclavicular joint, return to sport, shoulder injury, allograft, reconstruction, rehabilitation

## Abstract

**Background:** Acromioclavicular joint (ACJ) injuries are common in athletes, particularly in contact and collision sports, and frequently cause time lost from play. Although functional outcomes are well described, return to sport (RTS) is inconsistently reported, and the influence of treatment modality, surgical technique, and rehabilitation strategy on RTS outcomes remains uncertain. **Methods:** A systematic review was conducted following PRISMA guidelines registered in PROSPERO (ID 1155609). PubMed, Embase, Scopus, Web of Science, and Cochrane were searched for studies from 2015–2025 reporting at least one RTS metric (time, rate, or return to pre-injury level) after ACJ injury. Data on injury classification, surgical technique, rehabilitation protocols, and RTS outcomes were extracted. **Results:** Twenty-five studies (1077 patients) were included. The pooled RTS rate was 90.8% (95% CI, 88.6–93.0), with 87.7% (95% CI, 84.5–90.9) returning to their pre-injury level. The overall mean RTS time was 125.0 ± 63.05 days (4.5 months). Non-operative treatment was associated with faster RTS (52 days [95% CI, 47–58]) compared with operative management (127 days [95% CI, 114–140]). Among surgical techniques, allograft reconstruction demonstrated slightly higher rates of RTS at pre-injury level (84.2%) versus non-allograft approaches (78.9%). Rehabilitation timing was also influential: protocols initiating strengthening within 6 weeks were associated with faster RTS (93 vs. 132 days) and higher pre-injury RTS rates (86.8% vs. 72.7%). **Conclusions:** Most athletes return to sport after ACJ injury, with high RTS rates across treatment approaches. Earlier surgery, allograft reconstruction, and early strengthening show associative trends toward faster and more complete RTS, though these findings should be interpreted cautiously due to heterogeneity and confounding with existing data. Standardized RTS definitions, consistent rehabilitation reporting, and prospective comparative studies are needed to clarify which modifiable factors most influence recovery and return to play.

## 1. Introduction

Acromioclavicular joint (ACJ) injuries account for 9–11% of all shoulder injuries in the general population and up to 40–50% of shoulder injuries in athletes participating in contact sports such as football, rugby, ice hockey, and wrestling. The incidence among young athletes is approximately 9.2 per 1000 person-years, with the highest risk observed in males and those participating in collision sports [1,2,3]. Most injuries are low-grade sprains, but high-grade dislocations are more frequently seen in high-energy trauma and elite athletes. These injuries can result in pain, shoulder weakness, and impaired range of motion, directly affecting athletic performance. Average time lost from sport is ~10 days for low-grade sprains and ~64 days for high-grade injuries [2]. Nonoperative treatment generally allows athletes with low-grade injuries to return once pain-free with full strength and motion. In contrast, high-grade injuries often require surgical intervention, particularly in high-demand athletes, but surgery is associated with longer recovery, higher complication rates, and delayed return to sport [4,5]. Direct trauma is the most common mechanism, typically caused by a fall or blow to the lateral shoulder with the arm adducted. This is frequently seen in contact sports such as football, rugby, wrestling, and ice hockey, as well as cycling and alpine activities [6]. Indirect trauma is less common, resulting from a fall on an outstretched hand or elbow, with force transmitted to the ACJ, causing ligament sprain or separation. These injuries are more often lower grade but remain clinically relevant [7].

The Rockwood classification system describes six injury types ranging from a mild sprain without displacement (Type I) to complete disruption of both acromioclavicular and coracoclavicular ligaments with progressively severe displacement and soft-tissue injury (Types IV–VI). Management decisions are guided by this system: nonoperative care is recommended for Types I–II, surgery for Types IV–VI, and Type III injuries remain controversial [5].

ACJ injuries are disproportionately common in athletes, particularly those in contact and collision sports. They can significantly impair performance, delay return to play, and affect career longevity. While low-grade injuries often resolve quickly, higher-grade injuries are associated with prolonged recovery and inconsistent return-to-sport (RTS) criteria across the literature [8,9]. The greatest debate centers on Type III injuries, for which both operative and nonoperative management are used. Long-term functional outcomes are similar, but nonoperative care is often associated with faster early RTS and fewer complications, while surgery may offer improved alignment and cosmetic outcomes at the expense of hardware-related complications and delayed recovery [9,10,11]. Among surgical options (e.g., hook plate fixation, button devices, ligament reconstruction, etc.), none has demonstrated clear superiority in randomized trials [12,13,14].

Despite their high prevalence, there remains limited comparative data specifically addressing RTS timelines following ACJ injuries. Much of the existing literature emphasizes radiographic outcomes, complication rates, or long-term function, while athletes, coaches, and clinicians are often most concerned with how quickly and safely a return to preinjury level of performance can occur. Standardized RTS criteria are lacking, and reported recovery times vary considerably by injury severity, treatment modality, and sport-specific demands. Establishing clearer RTS benchmarks based on modifiable factors is critical to improving treatment decision-making, guiding athlete expectations, and informing evidence-based rehabilitation protocols. By defining recovery timelines more precisely, clinicians may also reduce reinjury risk and optimize return to high-performance levels.

The aim of this review is to summarize the existing literature on RTS after ACJ injury, building on a recent systematic review by Cleary et al. [8]. We specifically aim to compare RTS time, overall RTS rate, and return to pre-injury level across key modifiable factors, including Rockwood injury type, treatment modality, surgical technique, timing of surgery, and rehabilitation protocol. By organizing and analyzing these variables, this review seeks to clarify how modifiable factors may influence RTS outcomes and to identify trends that could inform clinical decision-making for athletes with ACJ injuries. Unlike prior reviews, this study provides a focused synthesis of modifiable, treatment-related, and rehabilitation-dependent factors influencing RTS, highlighting areas where standardization and prospective research are most needed. We hypothesize that while overall RTS rates will be high, consistent with prior literature, meaningful differences may exist across these modifiable factors.

## 2. Materials and Methods

### 2.1. Search Strategy

This review followed the PRISMA 2020 guidelines. We searched PubMed/MEDLINE, Embase, Scopus, Web of Science, and the Cochrane Library for studies published between 1 April 2015, and 1 April 2025. The Boolean search string used in PubMed was:

(acromioclavicular joint OR acromioclavicular OR “AC joint” OR ACJ OR “shoulder separation”) AND (“return to sport” OR “return to play” OR “athletic performance” OR sports OR athletes) AND (injury OR injuries OR trauma OR disruption OR dislocation).

This search string was adapted to the syntax of Embase, Scopus, Web of Science, and the Cochrane Library. Reference lists of included articles and relevant reviews were also screened. Grey literature and preprint servers were not searched. The final search was completed on 4 August 2025, and all results were compiled before screening began. Searches were performed using the above strategy, and all citations were uploaded to Rayyan (Rayyan Systems, Inc., Cambridge, MA, USA; version 1.4.3) for blinded screening.

This review was registered in the International Prospective Register of Systematic Reviews (PROSPERO; Registration ID: 1155609). PRISMA 2020 checklist is available in the Supplementary Materials (Table S1).

### 2.2. Selection Process

All search results were imported into Rayyan (version 1.4.3) for screening and deduplication. Two independent reviewers screened all titles and abstracts, followed by full-text review of potentially eligible studies. A calibration exercise was performed before screening to ensure consistency. Inter-rater agreement was measured using Cohen’s kappa. Disagreements were resolved by consensus or third-party adjudication. No automation tools beyond Rayyan’s deduplication function were used.

### 2.3. Eligibility Criteria

Inclusion criteria:

1. Clinical studies reporting on patients with ACJ injuries;
2. Reported RTS outcomes (time, rate, or return to pre-injury level);
3. Prospective or retrospective design;
4. Published in English.

Exclusion criteria:

1. Case reports, reviews, editorials, and conference abstracts;
2. Cadaveric or animal studies;
3. Studies without RTS outcomes;
4. Studies unavailable in full text.

A total of 25 studies were included in the final review.

### 2.4. Data Collection Process

Two reviewers independently extracted data into a standardized Excel spreadsheet (Microsoft Office 2024). Extracted data included study design, demographics, treatment modality, RTS criteria, rehabilitation protocol, and RTS outcomes. Discrepancies were resolved by consensus. Where needed, study authors were contacted for clarification.

### 2.5. Data Items

Variables extracted included study design, level of evidence, year of publication, country, sample size, demographics, competitive level of athletes, Rockwood classification, treatment modality (operative vs. non-operative), surgical technique, time to surgery, rehabilitation protocol, and RTS outcomes (time, rate, and pre-injury level). Complications, recurrence, and secondary surgeries were also collected when available. For rehabilitation timing, strengthening initiation was defined from surgery for operative cohorts and from injury for non-operative cohorts. Rehabilitation elements (immobilization duration, ROM milestones, and strengthening start) were extracted when available to illustrate protocol variability. There was variability in the definition of RTS criteria across the different studies, so RTS criteria was based on the ability of the athlete to return to athletic activity at any level after recovery from injury. Failure to RTS was defined as the athlete no longer participating in pre-injury athletic activities, which was largely based on patient reported data. Return to pre-injury level of play was defined as the ability of the athlete to perform athletic activity at the same level of performance as before the injury, which was again largely patient reported. Each study was analyzed for adequate RTS timelines, conditional criteria, and proper rehabilitation protocols to ensure accurate portrayal of RTS. Study specific RTS criteria and rehabilitation protocols are listed in the Supplementary Materials (Table S2). It is beyond the scope of this review to assess differences in RTS criteria across studies, and there is already an established need for more validated RTS guidelines after ACJ injury [8]. The data extraction methods and table design were partly adopted from the systematic review performed by Cleary et al. in 2024 [8].

### 2.6. Risk of Bias Assessment

Risk of bias was assessed independently by two reviewers. The Cochrane Risk of Bias 2 (RoB 2) tool was used for the single randomized controlled trial. The Newcastle–Ottawa Scale (NOS) was applied to all observational studies, including prospective and retrospective cohorts, comparative designs, and case series (with adapted criteria for case series). Disagreements were resolved by consensus.

Study-level risk assessments, including the tool applied and final rating (low, moderate, or high), are presented in the Supplementary Materials (Tables S3 and S4).

### 2.7. Certainty of Evidence Assessment

The overall certainty of evidence for the primary outcomes—RTS rate, pre-injury RTS rate, and mean time to RTS—was evaluated using the Grading of Recommendations, Assessment, Development, and Evaluation (GRADE) framework. Evidence was assessed across five domains: risk of bias, inconsistency, indirectness, imprecision, and publication bias. Because most included studies were observational with substantial heterogeneity in treatment and rehabilitation protocols, the certainty of evidence for all outcomes was rated as *low to very low*. A detailed GRADE summary is presented in the Supplementary Materials (Table S5).

### 2.8. Effect Measures

The primary outcome was time to RTS (days). Secondary outcomes were the RTS rate and the proportion of athletes returning to pre-injury level of play. Comparative effect measures (odds ratios, risk ratios, hazard ratios) with 95% confidence intervals were extracted where available.

### 2.9. Synthesis Methods

RTS outcomes were summarized descriptively across the included studies. For each study, we extracted the reported RTS rate (%), mean RTS time (days), and pre-injury RTS rate (%). When possible, the number of patients who returned to sport (n) and the total number of patients (N) were recorded, and pooled means and proportions were calculated using these aggregated n/N values rather than averaging percentages. This approach provided a more accurate descriptive representation of RTS outcomes across the literature.

Subgroup analyses were performed to explore potential influences of injury severity (Rockwood classification), treatment modality (operative vs. non-operative), surgical technique (allograft use), timing of surgery, and rehabilitation initiation (early vs. late). Because study definitions, rehabilitation protocols, and reporting standards varied widely, these analyses were presented descriptively to illustrate trends rather than to support statistical comparison.

Formal meta-analysis was not performed due to substantial clinical and methodological heterogeneity among studies. Exploratory heterogeneity assessment of the overall RTS data demonstrated I^2^ values ranging from 98.9% to 99.9%, confirming that the observed variability primarily reflected differences in study design, treatment approach, and patient population rather than random error. Therefore, a qualitative synthesis and pooled descriptive summary were used to characterize patterns in RTS outcomes. More detailed data for the heterogeneity assessment is included in the Supplementary Materials (Table S6).

### 2.10. Statistical Analysis

All data compilation and descriptive analyses were performed using Microsoft Excel (Microsoft Corporation, Redmond, WA, USA). Continuous variables, including RTS time, were reported as means ± standard deviations (SD) with 95% confidence intervals (CI) calculated using the normal approximation formula (mean ± 1.96 × SD/√n). Categorical variables (RTS rate, pre-injury RTS rate) were expressed as pooled proportions based on aggregated patient counts (n/N). Ninety-five percent CIs for proportions were calculated using the Wilson method. Because most included studies reported mean values rather than medians, pooled results were presented as means ± standard deviations for consistency across datasets. Given the reliance on mean values for RTS times, the underlying distributions may be skewed by outliers in the data; however, formal testing for normality could not be performed due to the use of study-level summary data. No inferential statistics or formal meta-analytic models were applied because of high heterogeneity in study design and data reporting across studies. Exploratory heterogeneity assessment for the overall RTS outcomes demonstrated I^2^ values between 98.9% and 99.9%, confirming substantial variability among studies. Therefore, all subgroup analyses were presented descriptively to illustrate general trends rather than statistical differences. Specific I^2^ values for each RTS outcome are listed in the Supplementary Materials (Table S6).

## 3. Results

### 3.1. Literature Search

**Figure 1:**
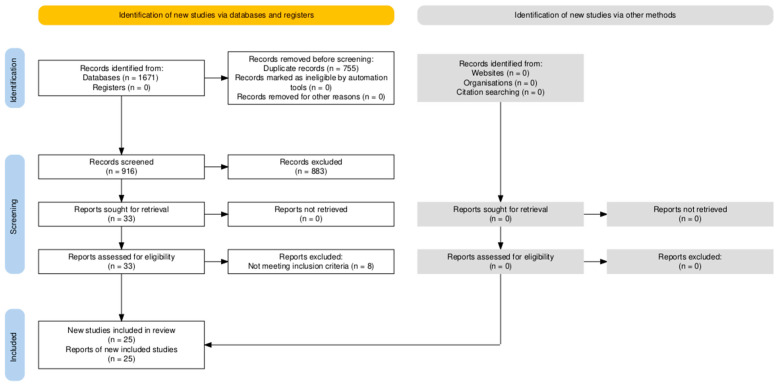
PRISMA (Preferred Reporting Items for Systematic Reviews and Meta-Analyses) study selection flow diagram.

The initial literature search resulted in 1671 total articles. After removal of duplicates, 916 article abstracts were screened for inclusion and exclusion criteria, with 33 unique studies passing initial screening and moving on to full text review for eligibility. After full texts were screened and evaluated, a total of 25 clinical studies were used for this review (Figure 1).

### 3.2. Study Characteristics and Patient Demographics

Our literature review found 25 studies that met our inclusion criteria, which included 1077 patients. The majority of patients were male (822/1077, 76.3%), with a mean age of 35.2 years ± 8.14 and mean follow-up of 35.4 months. A summary of study characteristics and patient demographics shown in Table 1.

### 3.3. Return to Sport

The overall rate of RTS was 90.8% (95% CI: 88.4–92.8), with 87.7% (95% CI: 84.1–90.7) of athletes returning to their pre-injury level of play. The overall mean RTS time was 125.0 ± 63.05 days (95% CI: 116.8–133.2), or 4.46 months, across all studies, which included all treatment modalities.

Definitions of RTS varied across studies and were based on differing criteria. To ensure transparency, a per-study summary of RTS definitions, criteria (time-based, functional, or clearance-based), and reported sport level has been included in the Supplementary Materials (Table S2).

### 3.4. Rockwood Classification

To assess the differences in RTS according to Rockwood classification, data was taken from studies that reported RTS outcomes and Rockwood injury type. This included 22/25 studies with 888 patients. The most common injury was Rockwood Type V (N = 416) and Rockwood Type III as the second most common (N = 283). Only 10 of the 22 studies reported RTS time by specific Rockwood class, which included 320 patients in total. Total patient counts and RTS data for all injury types are summarized in Table 2. One study included RTS data for Rockwood type I and II injuries, and the RTS rate was 100% with a mean RTS time of 20.1 days. The mean RTS time for Rockwood Type III, IV, and V were 82.7 ± 76.9 days (95% CI: 68.8–96.6), 122.1 ± 49.6 days (95% CI: 112.3–131.9), and 140.5 ± 27.3 days (95% CI: 134.7–146.3), respectively. There were no patients with a Rockwood Type VI injury. RTS rate was not uniformly reported across studies by specific Rockwood class, so individual rates could not be reported for each injury type.

### 3.5. Operative vs. Non-Operative

A total of 854 patients were treated operatively across 23 studies while 118 patients were treated non-operatively across 3 studies (Table 3). One study included two patient cohorts, one operative treatment and one non-operative treatment. RTS data reporting varied across different studies, so each data table includes the number of studies and patients that had available RTS data for each variable. The mean RTS time for operative patients was 126.8 ± 35.0 days (95% CI: 123.8–129.8) with a RTS rate of 91.3% (95% CI: 88.7–93.2), and the mean RTS time for non-operative patients was 51.7 ± 25.3 days (95% CI: 47.1–56.3) with a RTS rate of 87.5% (95% CI: 79.8–92.5). Seven studies also reported pre-injury level RTS data for operative patients, which had a mean of 81.2%. There was no pre-injury level RTS data available for the non-operative patients. All RTS data is summarized in Table 4, Table 5 and Table 6.

### 3.6. Surgical Technique

A total of 130 patients across 6 studies used allograft tendon as part of the reconstruction, and 729 patients across 23 studies did not use any allograft tendon as part of the reconstruction (Table 7). The allograft group had a mean RTS time of 129 ± 12.7 days (95% CI: 125.6–132.4), with a RTS rate of 88.0% (95% CI: 81.0–93.0) and pre-injury level RTS rate of 84.2% (95% CI: 62.4–94.5). The non-allograft group had a mean RTS time of 132.1 ± 39.5 days (95% CI: 128.7–135.5), with a RTS rate of 92.3% (95% CI: 89.6–94.3) and pre-injury level RTS rate of 78.9% (95% CI: 72.1–84.0). All RTS data summarized in Table 8, Table 9 and Table 10.

### 3.7. Time to Surgery

Time from injury to surgery was reported in 22 studies. Time to surgery was divided into an early surgery group, defined as receiving surgery within 21 days from injury, and a late surgery group that received surgery more than 21 days from injury. A total of 13 studies and 559 patients comprised the early surgery group, and there was a total of 9 studies and 239 patients for the late surgery group (Table 11). The early surgery group had a mean RTS time of 126.2 ± 35.1 days (95% CI: 123.1–129.3), with a RTS rate of 92.3% (95% CI: 89.1–94.7) and pre-injury level RTS rate of 84.1% (95% CI: 76.7–89.1). The late surgery group had a mean RTS time of 136.2 ± 47.8 days (95% CI: 128.3–144.1), with a RTS rate of 94.1% (95% CI: 90.2–96.5) and pre-injury level RTS rate of 68.9% (95% CI: 56.4–79.1). All RTS data summarized in Table 12, Table 13 and Table 14.

### 3.8. Rehabilitation Protocol

Post-injury rehabilitation protocols were described in 24 studies. Patients were divided into two groups based on whether strengthening rehab began prior to 6 weeks or after 6 weeks. For operative patients, the 6 weeks was measured from surgery date, while the 6 weeks was from injury date for non-operative patients. The early strengthening group included 307 patients across 10 studies, and the late strengthening group included 665 patients across 16 studies (Table 15). The early strengthening group had a mean RTS time of 93.3 ± 53.8 days (95% CI: 86.6–100.0) and RTS rate of 88.4% (95% CI: 84.1–91.7), whereas the late strengthening group had a mean RTS time of 132.4 ± 23.8 days (95% CI: 129.8–135.0) and RTS rate of 95.8% (95% CI: 93.3–97.5). Pre-injury level RTS rate for the early and late strengthening groups was 86.8% (95% CI: 77.4–92.7) and 72.7% (95% CI: 64.2–79.9), respectively. All RTS data is summarized in Table 16, Table 17 and Table 18.

## 4. Discussion

This systematic review evaluated RTS after ACJ injury, synthesizing data from 25 clinical studies and over 1000 patients. The key findings of this review are that overall RTS rates are high, with most athletes returning to pre-injury levels. This is consistent with prior systematic reviews [8,15,16,17,18,19], with RTS rates reported at 91–100%. The aim of this study was to synthesize reported data for ACJ injury with a focus on modifiable factors that affect RTS rates and RTS to pre-injury level of play. The overall RTS rate was high at 90.8%, with 87.7% of athletes regaining their pre-injury level of play, and a mean RTS time of approximately 4.5 months (125 days). These findings suggest that most athletes, regardless of treatment modality, can resume athletic participation after ACJ injury, although the results of this systematic review revealed some potential differences may exist based on injury severity, treatment approach, surgical timing, and rehabilitation strategy.

### 4.1. Rockwood Classification

Stratification by Rockwood classification revealed predictable trends in recovery, with RTS time increasing with greater injury severity. Type I and II injuries returned most quickly, while Types III–V demonstrated progressively longer recovery periods. These results are consistent with clinical practice patterns, in which higher-grade injuries require more extensive rehabilitation and, frequently, surgical stabilization [8,17,19]. However, many studies pooled RTS data across injury grades, limiting the ability to provide precise, grade-specific counseling. Even so, the observed gradient supports the prognostic value of the Rockwood classification not only for treatment selection but also for recovery timeline estimation.

### 4.2. Operative vs. Non-Operative Treatment

When comparing operative and non-operative management, notable differences emerged. Non-operative treatment was associated with a shorter mean RTS time (52 days) compared with operative treatment (127 days), while RTS rates were similar (87.5% vs. 91.3%). These findings align with prior evidence suggesting that non-operative care facilitates faster recovery in lower-grade injuries, whereas surgery is more commonly selected for higher-grade dislocations or athletes with high functional demands [9]. Importantly, only operative cohorts reported sufficient data on return to pre-injury level (81.2%), while such data was absent for non-operative patients. This reporting gap further emphasizes the importance of standardized outcome frameworks across both treatment modalities [8,9,17,19].

It should be noted that these findings may be confounded by injury severity. Operative management is typically selected for higher-grade dislocations, whereas non-operative care is favored for lower-grade injuries. Therefore, the observed difference in RTS time between the two groups may reflect baseline injury severity rather than a direct treatment effect. Because patient-level data was not available, we were unable to perform multivariable regression or stratified analysis to control for this confounding. Future meta-analysis incorporating individual patient data could clarify the independent contribution of treatment modality on RTS outcomes.

### 4.3. Surgical Technique

Surgical technique in this review was categorized based on whether tendon allograft or autograft was used versus primary repair without graft augmentation. This distinction was chosen for two main reasons. First, the included studies demonstrated considerable heterogeneity in fixation constructs (suspensory button devices, hook plates, coracoclavicular screws, and open versus arthroscopic techniques), making it difficult to meaningfully compare individual procedures. Grouping by graft use provided a unifying variable that cut across different fixation methods. Second, although graft augmentation is frequently debated in the surgical management of ACJ injuries, few studies have directly compared RTS outcomes between graft and non-graft techniques, leaving an important gap in the literature. Reporting of injury grade stratified by graft use was not consistent in the literature. In studies that provided grade information, cohorts in both graft and non-graft groups were predominantly high-grade (Rockwood III–V), and distributions were not reported in a manner that allowed grade-adjusted comparisons. Accordingly, observed differences should be interpreted descriptively rather than causally.

In our analysis, the effect of graft use on RTS outcomes was modest. Patients treated with allograft were associated with a slightly shorter mean RTS time (129 vs. 132 days) but a marginally lower RTS rate (88% vs. 92%). When these outcomes were considered together, no clinically significant differences were observed. One study suggested a higher pre-injury RTS rate in the graft group, though this was based on limited sample size and should be interpreted cautiously. These findings align with recent systematic reviews that showed no differences between open or arthroscopic techniques in loss of reduction and complication rates [20]. Cleary et al. [8] found that graft reconstruction was shown to have lower RTS times, with average RTS time of 3.6 months. RTS rates remained high across all surgical techniques, ranging from 86–98.5% in their study [8]. Among fixation constructs, cortical button techniques with multiple clavicular tunnels have consistently achieved excellent outcomes, suggesting that stability and surgeon familiarity may outweigh graft choice in predicting RTS.

Additionally, while our review found only modest differences in RTS outcomes between allograft and non-allograft reconstructions, it is notable that the allograft cohort was associated with a higher proportion of athletes returning to their pre-injury level of play (84.2% vs. 78.9%). This observation is consistent with growing evidence that allograft-based reconstructions may confer certain advantages in restoring sport-specific shoulder function. Biomechanical studies support this premise, showing that anatomic allograft reconstructions are associated with higher load-to-failure values and superior stability in both the vertical and horizontal planes compared to non-anatomic or suture-based techniques [21]. Such improvements in initial construct strength may allow for earlier progression through rehabilitation milestones and a safer, more confident return to sport.

Nonetheless, it should be emphasized that outcomes across graft types remain broadly comparable in the literature, and graft choice may ultimately be less important than achieving anatomic reconstruction with stable fixation [1,20]. Still, the consistently favorable RTS to pre-injury level observed with allograft reconstruction warrants further investigation, particularly in elite athletes where performance restoration is paramount.

Taken together, our findings indicate that graft use alone is unlikely to determine recovery trajectories. Future prospective studies, ideally randomized and standardized by athlete level and rehabilitation protocol, are needed to clarify the true impact of graft augmentation on RTS and functional outcomes.

### 4.4. Time to Surgery

The timing of surgical intervention appears to have an association with certain aspects of RTS after ACJ injury. In our analysis, early surgery (<21 days from injury) was associated with a shorter mean RTS time (126.2 vs. 136.2 days) and a higher rate of return to pre-injury level (84.1% vs. 68.9%) compared with delayed surgery, while overall RTS rates were similar between groups (92.3% vs. 94.1%). These findings suggest that although both early and late surgery allow for high rates of sport resumption, earlier intervention may optimize the likelihood of regaining pre-injury performance levels and reduce time away from competition.

There are several potential explanations for the findings in our review. Early stabilization may prevent the development of chronic soft tissue changes such as ligamentous scarring, muscle atrophy, and adaptive scapular dyskinesia, which can complicate surgical reduction and rehabilitation if treatment is delayed [22]. Acute repair of the ACJ capsule, ligaments, and deltotrapezial fascia within three weeks of injury allows for biological healing of soft tissues, whereas chronic cases require graft augmentation due to irreversible soft tissue changes. Delayed reconstruction has been associated with greater technical difficulty due to loss of anatomic landmarks, retraction of the coracoclavicular ligaments, and need for more extensive graft augmentation, all of which may contribute to longer recovery and reduced functional restoration [23].

Prior clinical studies have yielded mixed results [24,25,26]. Hazra et al. [24] reported that surgery within 21 days produced superior clinical outcomes compared with delayed intervention for Rockwood type III and V injuries, though RTS rates remained high in both groups. In contrast, Ladermann et al. [26] found no difference in long-term outcomes between early and delayed surgery when modern combined coracoclavicular and AC ligament reconstructions were used, though their definition of “delayed” often extended far beyond three weeks, limiting direct comparison to our study. Collectively, these findings suggest that while contemporary techniques may mitigate the disadvantages of delayed surgery, early intervention may still confer advantages, particularly for athletes seeking earlier return to pre-injury level.

Overall, RTS rates were high regardless of surgical timing, consistent with previous reviews [8,17]. However, the reduced likelihood of regaining pre-injury performance with delayed surgery highlights the potential benefit of early stabilization in competitive athletes and physically demanding populations. Shared decision making is essential to consider patient factors and desires to pursue the best decision possible. Future prospective studies with standardized RTS reporting and stratification by injury grade are needed to better define the role of surgical timing in optimizing sport-specific outcomes.

### 4.5. Rehabilitation Protocol

In our analysis, 23 of the 25 included studies (92%) reported rehabilitation protocols. Patients were stratified based on whether strengthening exercises began before or after 6 weeks post-surgery for operative patients and post-injury for non-operative patients. The early strengthening group included 307 patients across 10 studies, while the late strengthening group included 665 patients across 16 studies. Early strengthening was associated with a shorter mean RTS time (93.3 vs. 132.4 days), whereas the late strengthening group was associated with a slightly higher overall RTS rates (95.8% vs. 88.4%). Pre-injury RTS rates were higher in the early strengthening group (86.8% vs. 72.7%), suggesting that earlier initiation of controlled strengthening may facilitate faster functional recovery.

Rehabilitation is a critical determinant of RTS after ACJ injury, and protocols vary depending on treatment modality. Non-operative treatment generally allows early mobilization and progressive strengthening within 1–2 weeks once pain permits [4,6,27]. Post-operative rehabilitation typically begins with a period of immobilization, followed by staged progression from passive and active range of motion to strengthening. Historically, active isotonic strengthening was delayed until 12 weeks post-surgery due to concerns about construct tolerance to repetitive loads of force [27]. Recent literature, however, supports earlier initiation of strengthening once full range of motion is achieved, typically 6–8 weeks after surgery [6]. While prior systematic reviews have described rehabilitation protocols, few have directly compared RTS outcomes between different timing strategies [8,17].

There are several theoretical explanations for why early strengthening may accelerate RTS. The ACJ relies on both dynamic stabilizers (deltoid and trapezius muscles) and static stabilizers (capsule and ligaments) to maintain alignment and enable functional arm movements [6]. Early strengthening may enhance dynamic stability through neuromuscular control and hypertrophy of periarticular musculature, compensating for residual ligamentous laxity and optimizing biomechanics [6,21,27]. Additionally, early strengthening may prevent disuse atrophy and joint stiffness, facilitating restoration of full range of motion and strength symmetry—key criteria for safe RTS. Overall, these findings support the potential benefits of early controlled strengthening, but high-quality clinical studies are needed to define optimal timing, safety, and efficacy for athletes with ACJ injuries.

### 4.6. Limitations

This study has several limitations that should be acknowledged. First, the included literature was heterogeneous in terms of study design, injury classification, treatment approaches, rehabilitation protocols, and outcome reporting, which limited the ability to perform direct comparisons, statistical analysis, and meta-analysis. Many studies pooled patients with different Rockwood grades, precluding grade-specific conclusions. Second, the majority of included studies were retrospective in design with small sample sizes, introducing potential selection and reporting bias. Data on return to pre-injury level of sport was inconsistently reported, particularly in non-operative cohorts, restricting comparison across treatment modalities. Third, heterogeneity in surgical technique, including differences in fixation constructs, graft type, and surgeon experience, limited the ability to isolate the effect of individual procedures on RTS outcomes. Similarly, while most studies reported rehabilitation protocols, these were variably detailed and rarely standardized, making it difficult to fully account for their influence on recovery trajectories. Publication bias also cannot be excluded, as studies with favorable outcomes may be more likely to be published.

Finally, it is important to note that the factors examined in this study (such as timing of surgery, surgical technique, rehabilitation protocols, and injury grade) are not mutually exclusive and may interact with one another in complex ways. Controlling for these interdependencies was beyond the scope of this review. Comparisons across subgroups (e.g., operative vs non-operative, graft vs non-graft) are susceptible to confounding by injury severity, as higher-grade injuries are disproportionately represented in operative and allograft cohorts. Without access to individual patient data, statistical control for these covariates was not possible limiting causal inference. Therefore, observed differences within subgroups should be interpreted descriptively rather than causally. Because rehabilitation protocols and timing definitions were highly variable and incompletely reported, formal sensitivity analyses were also not feasible. Furthermore, the wide 95% confidence intervals calculated for pooled outcomes reflect substantial heterogeneity across studies and reinforce the descriptive intent of this review rather than inferential comparison. Instead, our aim was to summarize existing literature and highlight general trends for each factor individually, with the understanding that their combined influence may be more nuanced. These limitations underscore the need for well-designed prospective studies with standardized RTS reporting that can more directly evaluate the interplay of these factors and better define optimal treatment and rehabilitation strategies for athletes with ACJ injuries. According to the GRADE assessment, the overall certainty of evidence for all RTS outcomes was rated as low to very low, primarily due to observational study designs, heterogeneity in treatment and rehabilitation protocols, and inconsistent outcome definitions (Supplementary Table S6).

### 4.7. Future Directions

Future research should prioritize high-quality, prospective studies that use standardized definitions of RTS, including both overall return and return to pre-injury level of play. Randomized controlled trials comparing operative versus non-operative management, as well as different surgical constructs and graft techniques, are needed to clarify their relative impact on functional recovery. Equally important is the evaluation of rehabilitation protocols, particularly the timing and progression of strengthening, with a focus on safety and performance outcomes in athletes.

Importantly, future studies should also account for the interaction among key factors such as surgical timing, technique, rehabilitation protocols, and injury severity. These variables are not independent of one another, and their combined influence may determine RTS outcomes more than any single factor in isolation. Prospective study designs that stratify outcomes by Rockwood classification, sport-specific demands, and treatment timing will be critical for untangling these relationships and improving the precision of clinical recommendations.

Additionally, long-term follow-up is warranted to assess durability of repair, reinjury risk, and late complications such as post-traumatic arthritis or persistent instability. Finally, the adoption of standardized outcome reporting frameworks across ACJ injury studies would facilitate direct comparison and meta-analysis, enabling the development of evidence-based treatment and rehabilitation guidelines tailored to athletes.

## 5. Conclusions

Most athletes successfully return to sport after ACJ injury, with high overall RTS rates and generally favorable outcomes across treatment approaches. While earlier surgery, allograft reconstruction, and early rehabilitation were associated with trends toward faster and more complete return, these relationships remain uncertain due to heterogeneity in study design, injury severity, and rehabilitation protocols. The key finding of this review is the lack of standardization in how RTS outcomes and rehabilitation milestones are defined and reported. This variability, rather than the outcomes themselves, represents the main barrier to evidence-based consensus. Future research should prioritize consistent RTS definitions, transparent reporting of rehabilitation timing, and prospective comparative designs. Establishing these foundations will allow the field to determine which modifiable factors—such as surgical timing, graft selection, and rehabilitation progression—truly influence RTS performance after ACJ injury.

## Figures and Tables

**Table 1:** Study Characteristics and Patient Demographics.

	Value
Total patients, (n)	1077
Total studies, (n)	25
Level of Evidence, n (%)	
Level IV	14 (56)
Level III	9 (36)
Level II	1 (4)
Level I	1 (4)
Male sex, n (%)	822 (76.3)
Mean age ± SD (years)	35.1± 8.1 (range 22.4–59.9)
Mean Follow up ± SD (months)	35.4 ± 26.7 (range 0.9–134.2)

Note: Values are reported as mean ± standard deviation (SD).

**Table 2:** Rockwood Classification.

Rockwood Type	Number of Patients (n)	Number of Studies (n)	Return to Sport (Mean Days ± SD)	95% CI (Days)
I	7	1	20.1	Not calculable
II	11			
III	118	2	82.7 ± 76.9	68.8–96.6
IV	98	3	122.1 ± 49.6	112.3–131.9
V	86	4	140.5 ± 27.3	134.7–146.3
VI	0	0	NR	Not calculable
Total	320			

Values represent pooled means from 10 studies including 320 patients. Note: Values are reported as mean ± standard deviation (SD) or as pooled proportions (n/N). 95% confidence intervals (CI) represent patient-level pooled estimates calculated using the normal approximation for continuous data and the Wilson interval for proportions. NR = not reported.

**Table 3:** Operative vs. Non-operative.

Operative	
Patients (n)	854
Studies (n)	23
Non-operative	
Patients (n)	118
Studies (n)	3

Values represent pooled patient totals from 25 (1 study had both operative and non-operative groups) studies including 972 patients.

**Table 4:** Operative vs. Non-operative RTS Time.

	Return to Sport (days)	Studies (n)	Patients (n)	95% CI (days)
Operative	126.8 ± 35.0	13	525	123.8–129.8
Non-Operative	51.7 ± 25.3	3	118	47.1–56.3

Values represent pooled means from 16 studies including 643 patients. Note: Values are reported as mean ± standard deviation (SD) or as pooled proportions (n/N). 95% confidence intervals (CI) represent patient-level pooled estimates calculated using the normal approximation for continuous data and the Wilson interval for proportions.

**Table 5:** Operative vs. Non-operative RTS Rate.

	Return to Sport (%)	Studies (n)	Patients (n/N) *	95% CI (%)
Operative	91.3%	18	559/613	88.7–93.2
Non-Operative	87.5%	2	91/104	79.8–92.5

* n/N—n = number of patients who returned to sport/N = total number of patients. Values represent pooled proportions from 20 studies including 717 patients. Note: Values are reported as mean ± standard deviation (SD) or as pooled proportions (n/N). 95% confidence intervals (CI) represent patient-level pooled estimates calculated using the normal approximation for continuous data and the Wilson interval for proportions.

**Table 6:** Operative vs. Non-operative Pre-injury Level RTS Rate.

	Pre-Injury Return to Sport (%)	Studies (n)	Patients (n/N) *	95% CI (%)
Operative	81.2%	7	168/207	75.3–86.0
Non-Operative	NR	3 **	NR	Not Calculable

* n/N—n = number of patients who returned to sport/N = total number of patients. ** All 3 non-operative studies did not include data on pre-injury RTS rates. Values represent pooled proportions from 10 studies including 207 patients. Note: Values are reported as mean ± standard deviation (SD) or as pooled proportions (n/N). 95% confidence intervals (CI) represent patient-level pooled estimates calculated using the normal approximation for continuous data and the Wilson interval for proportions. NR = not reported.

**Table 7:** Surgical Technique.

**Allograft**	
Patients (n)	130
Studies (n)	6
**No Allograft**	
Patients (n)	729
Studies (n)	23

Values represent pooled patient totals from 25 (4 studies had both allograft and no allograft groups) studies including 859 patients.

**Table 8:** Surgical Technique RTS Time.

	Return to Sport (days)	Studies (n)	Patients (n)	95% CI (days)
Allograft	129 ± 12.7	2	53	125.6–132.4
No Allograft	132.1 ± 39.5	12	512	128.7–135.5

Values represent pooled means from 14 studies including 565 patients. Note: Values are reported as mean ± standard deviation (SD) or as pooled proportions (n/N). 95% confidence intervals (CI) represent patient-level pooled estimates calculated using the normal approximation for continuous data and the Wilson interval for proportions.

**Table 9:** Surgical Technique RTS Rate.

	Return to Sport (%)	Studies (n)	Patients (n/N) *	95% CI (%)
Allograft	88.0%	5	98/111	81.0–93.0
No Allograft	92.3%	13	467/506	89.6–94.3

* n/N—n = number of patients who returned to sport/N = total number of patients. Values represent pooled proportions from 18 studies including 617 patients. Note: Values are reported as mean ± standard deviation (SD) or as pooled proportions (n/N). 95% confidence intervals (CI) represent patient-level pooled estimates calculated using the normal approximation for continuous data and the Wilson interval for proportions.

**Table 10:** Surgical Technique Pre-injury Level RTS Rate.

	Pre-injury Return to Sport (%)	Studies (n)	Patients (n/N) *	95% CI (%)
Allograft	84.2%	1	16/19	62.4–94.5
No Allograft	78.9%	5	140/178	72.1–84.0

* n/N—n = number of patients who returned to sport/N = total number of patients. Values represent pooled means from 6 studies including 197 patients. Note: Values are reported as mean ± standard deviation (SD) or as pooled proportions (n/N). 95% confidence intervals (CI) represent patient-level pooled estimates calculated using the normal approximation for continuous data and the Wilson interval for proportions.

**Table 11:** Time to Surgery.

Early Surgery (<21 Days from Injury)	
Patients (n)	559
Studies (n)	13
Late Surgery (>21 days from injury)	
Patients (n)	239
Studies (n)	9

Values represent pooled patient totals from 22 studies including 798 patients.

**Table 12:** Time to Surgery RTS Time.

	Return to Sport (days)	Studies (n)	Patients (n)	95% CI (days)
Early Surgery	126.2 ± 35.1	12	498	123.1–129.3
Late Surgery	136.2 ± 47.8	5	142	128.3–144.1

Values represent pooled means from 17 studies including 640 patients. Note: Values are reported as mean ± standard deviation (SD) or as pooled proportions (n/N). 95% confidence intervals (CI) represent patient-level pooled estimates calculated using the normal approximation for continuous data and the Wilson interval for proportions.

**Table 13:** Time to Surgery RTS Rate.

	Return to Sport (%)	Studies (n)	Patients (n/N) *	95% CI (%)
Early Surgery	92.3%	10	325/352	89.1–94.7
Late Surgery	94.09%	8	207/220	90.2–96.5

* n/N—n = number of patients who returned to sport/N = total number of patients. Values represent pooled means from 18 studies including 572 patients. Note: Values are reported as mean ± standard deviation (SD) or as pooled proportions (n/N). 95% confidence intervals (CI) represent patient-level pooled estimates calculated using the normal approximation for continuous data and the Wilson interval for proportions.

**Table 14:** Time to Surgery Pre-injury Level RTS Rate.

	Pre-Injury Return to Sport (%)	Studies (n)	Patients (n/N) *	95% CI (%)
Early Surgery	84.1%	4	114/136	76.7–89.1
Late Surgery	68.9%	2	42/61	56.4–79.1

* n/N—n = number of patients who returned to sport/N = total number of patients. Values represent pooled means from 6 studies including 197 patients. Note: Values are reported as mean ± standard deviation (SD) or as pooled proportions (n/N). 95% confidence intervals (CI) represent patient-level pooled estimates calculated using the normal approximation for continuous data and the Wilson interval for proportions.

**Table 15:** Rehabilitation Protocol.

Strengthening ≤ 6 Weeks from Injury	
Patients (n)	307
Studies (n)	10
Strengthening ≥ 6 weeks from injury	
Patients (n)	665
Studies (n)	16

Values represent pooled means from 25 (1 study had both an early and late group) studies including 972 patients.

**Table 16:** Rehabilitation Protocol RTS Time.

	Return to Sport (days)	Studies (n)	Patients (n)	95% CI (days)
Strengthening ≤ 6 weeks	93.3 ± 53.8	8	246	86.6–100.0
Strengthening ≥ 6 weeks	132.4 ± 23.8	7	319	129.8–135.0

Values represent pooled means from 15 studies including 565 patients. Note: Values are reported as mean ± standard deviation (SD) or as pooled proportions (n/N). 95% confidence intervals (CI) represent patient-level pooled estimates calculated using the normal approximation for continuous data and the Wilson interval for proportions.

**Table 17:** Rehabilitation Protocol RTS Rate.

	Return to Sport (%)	Studies (n)	Patients (n/N) *	95% CI (%)
Strengthening ≤ 6 weeks	88.4%	8	245/277	84.1–91.7
Strengthening ≥ 6 weeks	95.8%	11	346/361	93.3–97.5

* n/N—n = number of patients who returned to sport/N = total number of patients. Values represent pooled means from 19 studies including 638 patients. Note: Values are reported as mean ± standard deviation (SD) or as pooled proportions (n/N). 95% confidence intervals (CI) represent patient-level pooled estimates calculated using the normal approximation for continuous data and the Wilson interval for proportions.

**Table 18:** Rehabilitation Protocol Pre-injury Level RTS Rate.

	Pre-injury Return to Sport (%)	Studies (n)	Patients (n/N) *	95% CI (%)
Strengthening ≤ 6 weeks from injury	86.8%	3	66/76	77.4–92.7
Strengthening ≥ 6 weeks from injury	72.7%	3	88/121	64.2–79.9

* n/N—n = number of patients who returned to sport/N = total number of patients. Values represent pooled means from 6 studies including 197 patients. Note: Values are reported as mean ± standard deviation (SD) or as pooled proportions (n/N). 95% confidence intervals (CI) represent patient-level pooled estimates calculated using the normal approximation for continuous data and the Wilson interval for proportions.

## Data Availability

The raw data supporting the conclusions of this article will be made available by the authors on request to corresponding author.

## Supplementary Materials

The following supporting information can be downloaded at: https://www.mdpi.com/article/10.3390/jcm14217656/s1, Table S1: PRISMA 2020 Checklist; Table S2: Return To Sport Definitions & Rehabilitation Protocols; Table S3: RoB 2 Risk of Bias Table (RCTs); Table S4: NOS Risk of Bias (Observational Studies); Table S5: Certainty of Evidence for Primary Return-to-Sport Outcomes (GRADE Framework); Table S6: Pooled Return to Sport (RTS) Data. Ref. [28] is cited in Table S1.
