# Supplementary material for: Return to Sport After Acromioclavicular Injury: A Systematic Review of Modifiable Factors
Source: J Clin Med. 2025 Oct 28;14(21):7656. doi: 10.3390/jcm14217656 (PMC12608991; doi:10.3390/jcm14217656)
Supplement: Supplementary file 1 [file jcm-14-07656-s001.zip › Tables S2-S6.pdf]

Table S2. Return To Sport Definitions & Rehabilitation Protocols

Title	Return to Sport Definition	Rehabilitation Protocol
Coracoclavicular Space Widening on Radiographs After Arthroscopic Stabilization With Suspensory Fixation Does Not Affect Athletic Performance	Self-reported resumption of participation in their sport following the standardized rehabilitation protocol.	All patients must have completed 6 months after the fixation and had the same rehabilitation program, which was formulated in 4 phases over 6 months, including:<br>(1) immobilization phase (weeks 0- 6): during this phase, all patients were immobilized in a pouch arm sling<br>(2) intermediate phase (weeks 7-12): during this phase, active assisted and active range of motion were initiated along with stretching exercises<br>(3) strengthening phase (weeks 13-16); during this phase, strengthening exercises including isotonic strength activities were initiated<br>(4) return to activity phase (months 4-6); during this phase, strength, balance and proprioception exercises, and sports-specific activities were started.
Return to sport after acute acromioclavicular stabilization: a randomized control of double-suture-button system versus clavicular hook plate compared to uninjured shoulder sport athletes	Resumption of preinjury athletic participation evaluated longitudinally (6, 12, and 24 months) using ASOSS, SSAS, and NAS scores.	Clavicular hook plate group:<br>**Postoperative week 0–3:**<br>Brace: Gilchrist for pain relief (3–5 days)<br>Stress: passive-assisted physiotherapy, No weight bearing<br>ROM: passive ABD/FLEX to 60°<br>**Post-operative week 4–6:**<br>Brace: no<br>Stress: active-assisted physiotherapy, No weight bearing<br>ROM: passive ABD/FLEX to 90°<br>**Post-operative week 7–12:**<br>Brace: no<br>Stress: active-assisted physiotherapy, No weight bearing<br>ROM: ABD/FLEX to 90°<br>**Post-operative week 8–10:**<br>2nd Surgery: hook plate removal<br>**Post-operative month 4–6:**<br><br>Double-suture-button group:<br>**Postoperative week 0–3:**<br>Brace: SAS 15° day and night<br>Stress: passive-assisted physiotherapy, No weight bearing<br>ROM: passive ABD/FLEX to 45°<br>**Post-operative week 4–6:**<br>Brace: no<br>Stress: active-assisted physiotherapy, No weight bearing<br>ROM: passive ABD/FLEX to 90°<br>**Post-operative week 7–12:**<br>Brace: no<br>Stress: active-assisted physiotherapy, No weight bearing

		Brace: no<br>Stress: active physiotherapy<br>ROM: free<br>Important: fitness sport; no contact<br>**Post-operative month 7–10:**<br>Brace: no<br>Stress: active physiotherapy with full stress<br>ROM: free<br>Important: No competition sport<br>**Post-operative from month 10:**<br>Free	ROM: ABD/FLEX to 90°<br>**Post-operative month 4–6:**<br>Brace: no<br>Stress: active physiotherapy<br>ROM: free<br>Important: fitness sport; no contact<br>**Post-operative month 7–10:**<br>Brace: no<br>Stress: active physiotherapy with full stress<br>ROM: free<br>Important: No competition sport<br>**Post-operative from month 10:**<br>Free
Acromioclavicular Joint Separation in UEFA Soccer Players: A Matched-Cohort Analysis of Return to Play and Player Performance From 1999 to 2018	Return to elite competition (i.e., participation in official matches in the top 5 European soccer leagues).	Not Reported	
Acromioclavicular Joint Injuries in Professional Ice Hockey Players: Epidemiologic and MRI Findings and Association With Return to Play	Criteria for return-to-play approval included objective measures of manual strength with a handheld dynamometer and Y-balance testing compared with preinjury baseline testing, functional assessments of push-up and resisted push-pull testing, and ability during practice to receive contact, battle with stick, and shoot confidently. Manual strength measures were made relative to baseline (preseason) measures of the same extremity, with	All grade 1, grade 2, and grade 3 ACJ injuries (n= 23) were treated with nonoperative management, including pain control and immediate postinjury support with a shoulder sling, followed by early mobilization and progressive resistance exercises followed by strength and physical reconditioning.	

	goals of 90% return to baseline before clearance to return to play.	
A retrospective comparative study of arthroscopic fixation in acute Rockwood type IV acromioclavicular joint dislocation: single versus double paired Endobutton technique	Resumption of the patient's preinjury sporting activities following surgical fixation and standardized rehabilitation.	After surgery, a sponge shoulder abduction orthosis was used to fix the shoulder joint at 0-degree external rotation position and was kept for 6 weeks. Forty-eight hours after surgery, all patients were encouraged to participate in the following rehabilitation program: passive abduction, forward flexion, and external rotation with the arm at the pain tolerance level. Active movements for the arm began at 6 weeks post operation. Normal activities, daily work, and limited rehab sports were allowed at 3 months after surgery. No difference in the rehabilitation program was noted between the two groups.
Clinical outcomes of a single-tunnel technique for coracoclavicular and acromioclavicular ligament reconstruction	Resumption of preinjury athletic participation following completion of the structured rehabilitation program and physician clearance.	Postoperatively, the affected arm was immobilized in a sling with an abduction pillow for 1 month. For the first month, only passive range of motion was allowed. Over the next 4 weeks, patients progressed to active range of motion, as long as full passive range of motion was obtained. After another 4 weeks, if full active motion was obtained, patients were allowed to start strengthening. This was all done under the supervision of a licensed physical therapist. Patients were then prepared to return to their sports using sports-specific training. Clearance to return to contact sports was usually given between 6 and 9 months.
Surgical treatment of acute type V acromioclavicular joint dislocations in professional athletes: an anatomic ligament reconstruction with synthetic implant augmentation	Self-reported resumption of the patient's preinjury level of sports participation.	A broad arm sling for a week aided comfort, after which gradual mobilization was encouraged. Physiotherapy was not usually necessary, and full movement was gradually regained at 6 weeks. The patients were informed not to exceed the prescribed activity levels or to overload the repair before the completion of 3 months after operation. All mobilization and exercises were performed within the pain-free range of movement. A gradual buildup of strength during the next few months allowed return to full activity. All athletes were allowed to return to sport without restrictions at 6 months.

Open capsular and ligament reconstruction with semitendinosus hamstring autograft successfully controls superior and posterior translation for type V acromioclavicular joint dislocation	Self-reported resumption of the patient's preinjury level of sports participation.	The arm of the operated shoulder was immobilized in a sling with abduction pillow for 6 weeks. No shoulder motion was allowed for 3 weeks. Supine passive motion in the scapular plane is begun at 3 weeks (forward flexion to 90°, full external rotation, and no internal rotation). Active motion begins at week 7. Resistance exercises begin at week 12. Return to full activity including contact sports is allowed at week 16.
Sports activity after anatomic acromioclavicular joint stabilization with flip-button technique	Resumption of the patient's preinjury sport activity, including type, frequency, and intensity was collected via patient questionnaire (provided in appendix of study article).	The post-operative rehabilitation protocol consisted of immobilization of the arm in a shoulder abduction orthosis (Medi SAK®; Medi, Bayreuth, Germany) at 30° for 4 weeks. Pain-free, passive range of motion up to 90° abduction and flexion and 40° internal/external rotation (IR/ER) was started on the first day post-surgery. Active motion restricted at 90° abduction and flexion and 40° IR/ ER rotation was allowed after four weeks. Active range of motion on all ranges was allowed after 6 weeks, but overhead motion was especially trained under supervision of a physiotherapist at the start. Strength training for rotator cuff, deltoid and scapula stabilizers was gradually increased beginning at 6 weeks post-operative. Three months after surgery carrying weight and non-contact sports were allowed. After 6 months, the patients were allowed to return to sports without restriction.
Comparing mini-open and arthroscopic acromioclavicular joint repair: functional results and return to sport	Self-reported measures of athletic performance using SPORTS score	All patients used Kenny Horward or Desault sling for a period of 4 weeks. They did not return to play sports until 6 weeks.
Return-to-activity after anatomical reconstruction of acute high-grade acromioclavicular separation	Participation in sporting activities following arthroscopically assisted anatomical AC joint reconstruction for acute Rockwood Type V separation.	Initial postoperative treatment consisted of limited ROM under instruction of a physical therapist and immobilization of the upper extremity with a standard arm sling for 6 weeks for protection. Afterwards free ROM was allowed, unlimited activities of daily living were advised not before 12 weeks and return to overhead and/or contact sports not earlier than 6 months after surgery.
Arthroscopic treatment of acute acromioclavicular dislocation using a double button device: Clinical and MRI results	Resumption of sport participation according to type of sport (using	Patients were given a standard surgical corset immediately after surgery. Passive range of

	Allain's classification, G1–G4 from low to high demand).	motion and pendulum exercises were started one week postoperatively and conducted over a period of six weeks. Then the corset was removed, and patients started active range of motion and strength exercises. They were told to abstain from lifting, carrying, pushing, and pulling on the operated side for a period of three months after the surgery.
More Than 90% Satisfactory Results After Non-Surgical Treatment of Type III and V Acute AC Joint Dislocation: A Prospective Cohort Study With 1-Year Follow-up of 95 Patients Managed Non-Surgically with the Option of Delayed Surgical Intervention	Resumption of preinjury sport participation after non-surgical management.	Not Reported.
Arthroscopic Coracoclavicular Ligament Reconstruction with Double-Bundle Soft Tissue Allograft for Chronic Type V Acromioclavicular Dislocations: Patient Outcomes and Return to Duty and Sport at Minimum 10-year Follow-Up	Self-reported resumption of sport participation	All patients were placed in a gunslinger brace for six weeks and instructed to perform elbow range of motion exercises. Rehabilitation progressed to active and passive shoulder range of motion after six weeks. From six to 12 weeks, patients progressed through a graduated rehabilitative course initially focused on motion and normal shoulder kinematics. Strengthening began 12 weeks postoperatively and return to contact or high-fall-risk activities was permitted after 6 months.
High Vertical and Horizontal Stability at Short-Term Follow-Up After an All-Endoscopic Double Cerclage Endobutton Technique for Acute Acromioclavicular Joint Separations	Return to sport is determined individually and in an interdisciplinary manner with the physiotherapist, trainer, and patient.	Postoperatively, patients are immobilized in a 30° abduction shoulder sling for 6 weeks. Pendulum exercises and passive, pain-free mobilization are permitted. Active rehabilitation is performed after 6 weeks.
Arthroscopically Assisted Acromioclavicular Joint Stabilization in Patients Aged ≥50 Years Results in a Low Rate of Clinical Failure, Favorable Outcomes, and High Return to Activity and Work	The time to return to activity, the time to return to the current level of athletic activity, and a subjective change in sporting ability (ordinal scale consisting of "improved," "equal to preoperatively," or "deteriorated") were assessed via patient questionnaire.	The operated arm was immobilized in a sling for 6 weeks. Limited active-assisted range-of-motion exercises were gradually increased over 6 weeks. Active full range-of-motion exercises were started after 6 weeks, followed by a gradual return to overhead activities with load after 12 weeks, after clinical and radiographic control. Patients were advised to wait for 6 months before returning to full-contact sports. Return to work was coordinated in accordance with the return-to sports protocol. Rehabilitation protocols did not differ based on etiology.
A combined technique for acromioclavicular reconstruction after acute dislocation – technical description and functional outcomes	Self-reported resumption of patient prior level of sport or occupational	Initially the shoulder is immobilized with a sling, allowing pendular shoulder movements. Around the 4–6th week K-wires are extracted.

	activity following surgical reconstruction.	Until then we limit active shoulder elevation and abduction to 90°.
Acromioclavicular joint separation: Retrospective study of non-operative and surgical treatment in 38 patients with grade III or higher injuries and a minimum follow-up of 1 year	Self-reported resumption of sport participation after treatment for AC joint separation	**Operative:**<br>The operated arm was immobilized postoperatively in a shoulder immobilizer with the elbow internally rotated at the side for 4 to 6 weeks. After this immobilization period, a rehabilitation specialist was tasked with helping the patient progress from passive to active-assisted to active range of motion, two to three times per week plus self-directed rehabilitation at home. Muscle strengthening was started only in the 3rd month postoperative.<br>**Non-operative:**<br>Non-operative treatment consisted of providing the patients with a sling to relieve pain (length of immobilization was not defined and left up to the patient). Physical therapy was not always prescribed.
Hybrid coracoclavicular and acromioclavicular reconstruction in chronic acromioclavicular joint dislocations yields good functional and radiographic results	Return to sports was allowed 6 months after surgery following a radiographic and clinical assessment.	The patients wore a sling for 6 weeks postoperatively. Gradual recovery of the passive range of motion (ROM) was then initiated and continued until full passive elevation was achieved. Active ROM and muscle strengthening exercises were allowed once the passive ROM was fully recovered to avoid scapula-thoracic imbalance. Return to sports was allowed 6 months after surgery following a radiographic and clinical assessment.
Long-Term Functional Outcomes and Athletic Ability in Shoulder Sports After Anatomic Coracoclavicular Ligament Reconstruction for Chronic Type 3 and 5 Acromioclavicular Joint Injuries	Resumption of preinjury sporting activity, measured using the Subjective Patient Outcome for Return to Sports (SPORTS) score (0–10 scale).	Postoperative management was performed using a shoulder unloader brace (Lerman Shoulder Orthosis; DonJoy) for 6 weeks. This allowed for the unloading of the shoulder to avoid stress being placed on the ACJ reconstruction. Patients were allowed to initiate upright range of motion exercises 2 months after surgery. If the patient was pain-free, strengthening exercises were implemented after 12 weeks, focusing on scapular stabilizers to reduce ACJ loads. Weight training was incorporated within 3 to 5 months and return to contact athletic activity began as early as 6 months after surgery.
Mid-term clinical and sonographic outcomes of minimally invasive acromioclavicular joint	Patient reported resumption of sporting activity via questionnaire (0: no	Postoperative treatment was administered according to a standard protocol after ACJ

reconstruction: mini-open versus arthroscopically assisted	sports possible–4: return to pre-injury sport level).	stabilization. All patients were treated with a brace immediately after surgery for 6 weeks. Active-assisted range of motion up to 60° of abduction/flexion was allowed immediately after surgery for 3 weeks and up to 90° in the following 3 weeks. Afterward, range of motion was unlimited, but muscle strengthening exercises were paused up to 10–12 weeks. Competitive athletes and patients with a high functional demand were first allowed to return to sports 3 months after surgery.
Mini-open vs. arthroscopic double tight-rope reconstruction after acute AC-joint dislocation: a comparison in functional outcome and sports activity	Assessed using two validated grading systems:<br><br>Valderrabano Sports Activity Level – quantifying overall sport participation frequency (1–5 scale: 1 = none, 5 = competitive sport).<br><br>Rhee Recovery of Athletic Activity Score – grading recovery relative to preinjury level (1–5 scale: 1 = full recovery, 5 = no sport possible).	The postoperative recovery program was identical in both groups. An arm sling (Medi © Armschlinge) was used to ensure stability of the joint and maintain the correct position of the implant, which the patients were to wear throughout the first 4 weeks following the surgical procedure. Additionally, patients received an ossification prophylaxis with celecoxib given at a dose of 200 mg twice daily for 2 weeks after the surgery. Contracture prophylaxis was administered through physiotherapeutic assisted mobilization of the shoulder in abduction and flexion up to 40 degrees until completion of the second postoperative week. Thereafter, passive extension of the mobility range was increased to 90 degrees. Full stress of the joint as well as weight-bearing on the extended arm and rotational movement of the shoulder were to be avoided until after the sixth postoperative week. Neither one of the applied surgical techniques required the removal of materials after recovery.
Posterior Distal Clavicle Beveling for Chronic Nonincarcerated Type IV Acromioclavicular Separations: Surgical Technique and Early Clinical Outcomes	Self-reported resumption of the patient's preinjury athletic activity following surgery via survey.	Postoperative treatment was similar to that of an arthroscopic acromioplasty in which the patient was requested to discontinue the use of the sling 2 days after the surgery. Patients began a physical therapy program approximately 1 week after surgery, focusing on scapular stabilization and progressive rotator cuff strengthening, avoiding adduction exercise.
Return to Play After Surgical Treatment of High-Grade Acromioclavicular Joint Injuries in the Australian Football League	Return to play was defined as returning to a scheduled competitive match at the same level that was played by the athlete before surgery.	The postoperative rehabilitation protocol included immobilization in a neutral sling only until wound healing. Passive and active assisted mobilization was allowed without limitation from day 1 postoperatively and removal of the sling for active movement from 1 week

		postoperatively. No weightbearing or strengthening exercises were allowed on the affected extremity before 3 weeks. Return to noncontact training was permitted after 4 weeks, and contact (collision) training if the player had adequate shoulder strength after 5 weeks. Players were cleared to full-contact training and play from 6 weeks onward.
Single tunnel technique versus coracoid sling technique for arthroscopic treatment of acute acromioclavicular joint dislocation	Self-reported resumption of their previous level of sports and activity.	The same rehabilitation program was utilized in both groups. The shoulder joint was placed in a sling sponge shoulder abduction immobilizer at 0° external rotation position for 6 weeks postop. From the second postoperative day onwards, passive Gentle pendulums and Codman's were encouraged depending on the patients' pain tolerance level. Patients were instructed to not resume active movement of the arm until 6 weeks post-op. Patients were generally allowed to return to normal activities and daily work but limited sports until 3 months post-op depending on the level of rehabilitation. In addition, they were asked not to engage in contact sports prior to six months post-op.

Table S3. RoB 2 Risk of Bias Table (RCTs)

Title	Study Design	Level of Evidence	Risk of Bias Rating
Coracoclavicular Space Widening on Radiographs After Arthroscopic Stabilization With Suspensory Fixation Does Not Affect Athletic Performance	Prospective nonrandomized comparative trial	III	Low to Moderate
Return to sport after acute acromioclavicular stabilization: a randomized control of double-suture-button system versus clavicular hook plate compared to uninjured shoulder sport athletes	Prospective randomized controlled trial (64.4% randomized, remainder intention-to-treat)	II	Low to Moderate

Table S4. NOS Risk of Bias (Observational Studies)

Title	Study Design	Level of Evidence	Risk of Bias Rating
Acromioclavicular Joint Separation in UEFA Soccer Players: A Matched-Cohort Analysis of Return to Play and Player Performance From 1999 to 2018	Retrospective cohort; matched 2:1 controls; Level of evidence 3	III	High
Acromioclavicular Joint Injuries in Professional Ice Hockey Players: Epidemiologic and MRI Findings and Association With Return to Play	Retrospective case series	IV	High
A retrospective comparative study of arthroscopic fixation in acute Rockwood type IV acromioclavicular joint dislocation: single versus double paired Endobutton technique	Retrospective comparative study	III	Moderate

Clinical outcomes of a single-tunnel technique for coracoclavicular and acromioclavicular ligament reconstruction	Retrospective case series	IV	High
Surgical treatment of acute type V acromioclavicular joint dislocations in professional athletes: an anatomic ligament reconstruction with synthetic implant augmentation	Case series	IV	High
Open capsular and ligament reconstruction with semitendinosus hamstring autograft successfully controls superior and posterior translation for type V acromioclavicular joint dislocation	Retrospective case series	IV	High
Sports activity after anatomic acromioclavicular joint stabilisation with flip-button technique	Retrospective case series	IV	High
Comparing mini-open and arthroscopic acromioclavicular joint repair: functional results and return to sport	Retrospective cohort	Not reported	High
Return-to-activity after anatomical reconstruction of acute high-grade acromioclavicular separation	Retrospective clinical study (case series)	IV	High
Arthroscopic treatment of acute acromioclavicular dislocation using a double button device: Clinical and MRI results at 2 years	Retrospective case series	IV	High
More Than 90% Satisfactory Results After Non-Surgical Treatment of Type III and V Acute AC Joint Dislocation: A Prospective Cohort Study With 1-Year Follow-up of 95 Patients Managed Non-Surgically with the Option of Delayed Surgical Intervention	Prospective cohort study	Not reported	Moderate
Arthroscopic Coracoclavicular Ligament Reconstruction with Double-Bundle Soft Tissue Allograft for Chronic Type V Acromioclavicular Dislocations: Patient Outcomes and Return to Duty and Sport at Minimum 10-year Follow-Up	Retrospective review of prospectively collected cohort	IV	Moderate
High Vertical and Horizontal Stability at Short-Term Follow-Up After an All-Endoscopic Double Cerclage Endobutton Technique for Acute Acromioclavicular Joint Separations	Retrospective comparative clinical series	III	Moderate
Arthroscopically Assisted Acromioclavicular Joint Stabilization in Patients Aged ≥50 Years Results in a Low Rate of Clinical Failure, Favorable Outcomes, and High Return to Activity and Work	Retrospective outcome study of prospectively collected data	IV	High
A combined technique for acromioclavicular reconstruction after acute dislocation – technical description and functional outcomes	Observational retrospective study	Not reported	High
Acromioclavicular joint separation: Retrospective study of non-operative and surgical treatment in 38 patients with grade III or higher injuries and a minimum follow-up of 1 year	Retrospective two-center study	III	High
Hybrid coracoclavicular and acromioclavicular reconstruction in chronic acromioclavicular joint dislocations yields good functional and radiographic results	Retrospective case series	IV	High
Long-Term Functional Outcomes and Athletic Ability in Shoulder Sports After Anatomic Coracoclavicular Ligament Reconstruction for Chronic Type 3 and 5 Acromioclavicular Joint Injuries	Case series	IV	High

Mid-term clinical and sonographic outcomes of minimally invasive acromioclavicular joint reconstruction: mini-open versus arthroscopically assisted	Retrospective two-center comparative study	III	Moderate
Mini-open vs. arthroscopic double tight-rope reconstruction after acute AC-joint dislocation: a comparison in functional outcome and sports activity	Retrospective comparative study	I	Moderate
Posterior Distal Clavicle Beveling for Chronic Nonincarcerated Type IV Acromioclavicular Separations: Surgical Technique and Early Clinical Outcomes	Retrospective case series	IV	High
Return to Play After Surgical Treatment of High-Grade Acromioclavicular Joint Injuries in the Australian Football League	Retrospective case series	IV	High
Single tunnel technique versus coracoid sling technique for arthroscopic treatment of acute acromioclavicular joint dislocation	Retrospective comparative study	III	Moderate

Table S5: Certainty of Evidence for Primary Return-to-Sport Outcomes (GRADE Framework)

Outcome	Risk of Bias	Inconsistency	Indirectness	Imprecision	Publication Bias	Overall Certainty
Return-to-Sport (RTS) Rate	Serious	Serious	Not serious	Serious	Likely	Low
Return-to-Pre-Injury RTS Rate	Serious	Serious	Not serious	Serious	Likely	Very Low
Time to RTS	Serious	Serious	Not serious	Serious	Possible	Low

Certainty of evidence was evaluated using the GRADE (Grading of Recommendations, Assessment, Development, and Evaluation) framework. Evidence was downgraded due to observational study designs, heterogeneity in rehabilitation and treatment protocols, inconsistent outcome definitions, and small sample sizes. Overall certainty was rated as low to very low across all outcomes. Abbreviations: RTS = return to sport; CI = confidence interval.

Table S6: Pooled Return to Sport (RTS) Data

Variable	Studies (N)	Mean	SD	I^2 (%)
RTS rate	21	90.8%	9.8	98.9
RTS time (days)	18	125	63.1	99.97
Pre-injury RTS rate	17	87.7%	11.7	99.3
